# YY1/BCCIP Coordinately Regulates P53-Responsive Element (p53RE)-Mediated Transactivation of p21^Waf1/Cip1^

**DOI:** 10.3390/ijms20092095

**Published:** 2019-04-28

**Authors:** Yi Sui, Tingting Wu, Fuqiang Li, Fei Wang, Yong Cai, Jingji Jin

**Affiliations:** 1 Department of Biochemistry and Molecular Biology, School of Life Sciences, Jilin University, Changchun 130012, China; suiyi0910325@163.com (Y.S.); ttwu18@mails.jlu.edu.cn (T.W.); fei@jlu.edu.cn (F.W.); 2 School of Pharmacy, Changchun University of Chinese Medicine, Changchun 130117, China; lifuqiang157@163.com; 3 National Engineering Laboratory for AIDS Vaccine, School of Life Sciences, Jilin University, Changchun 130012, China; 4 Key Laboratory for Molecular Enzymology and Engineering, the Ministry of Education, Jilin University, Changchun 130012, China

**Keywords:** transactivation, gene transcription, p53, BCCIP, *p21*, YY1

## Abstract

Transactivation of *p21* (cyclin-dependent kinase inhibitor 1A, CDKN1A) is closely related to the recruitment of transcription cofactors at the p53 responsive elements (p53REs) in its promoter region. Human chromatin remodeling enzyme INO80 can be recruited to the p53REs of *p21* promoter and negatively regulates *p21*. As one of the key subunits of the INO80 complex, YY1 has also been confirmed to bind to the p53RE sites of *p21* promoter. Importantly, YY1 was recently reported to be bound and stabilized by BCCIP (BRCA2 and CDKN1A-interacting protein). Therefore, we hypothesized that the YY1/BCCIP complex plays an important role in regulating the transactivation of *p21*. Here we present evidence that the YY1/BCCIP complex coordinatively regulates p53RE-mediated *p21* transactivation. We first confirmed the cross-interaction between YY1, BCCIP, and p53, suggesting an intrinsic link between three proteins in the regulation of *p21* transcription. In dual luciferase assays, YY1 inhibited p53RE-mediated luciferase activity, whereas BCCIP revealed the opposite effect. More interestingly, the region 146–270 amino acids of YY1, which bound to BCCIP, increased p53-mediated luciferase activity, indicating the complexity of the YY1/BCCIP complex in co-regulating *p21* transcription. Further in-depth research confirmed the co-occupancy of YY1/BCCIP with p53 at the p53RE-proximal region of *p21*. Lentiviral-mediated knockdown of BCCIP inhibited the recruitment of p53 and YY1 at the p53RE proximal region of *p21*; however, this phenomenon was reversed by expressing exogenous YY1, suggesting the collaborative regulation of YY1/BCCIP complex in p53RE-mediated *p21* transcription. These data provide new insights into the transcriptional regulation of *p21* by the YY1/BCCIP complex.

## 1. Introduction

The *TP53* gene encodes the p53 protein that plays critical role in tumor prevention by taking control of a wide variety of cellular responses and the expression of multiple genes that regulates stress signal pathways [1]. In cancer cells, p53 is usually degraded and therefore becomes inactive [2]. However, p53 is activated upon cellular stresses such as DNA damage and transcriptionally activates sets of genes to play a role in DNA repair, cell cycle arrest, and apoptosis [3]. Structural research analysis shows that p53 consists of 393 amino acids and is composed of three distinct functional domains: (i) an N-terminal domain (1–93 amino acids) containing a transcriptional activation domain and a proline-rich domain; (ii) a core DNA-binding domain (102–292 amino acids), which contains most of the inactivating mutations found in human tumors; and (iii) a C-terminal domain consisting of a tetramerization domain (320–356 amino acids) and regulatory domain (363–393 amino acids). Among them, the DNA binding domain is well structured. In contrast both the N- and C-terminal domains are intrinsically disordered [4,5]. These different domains can be bound by different proteins, demonstrating the diversity of the biological functions of p53. For example, the co-activator p300-dependent acetylation of the C-terminal domain of p53 can stabilizes the protein by preventing Mdm2-mediated degradation [6].

It is well known that CDKN1A (*p21*^Waf1/Cip1^, *p21*) is a universal inhibitor of cyclin kinases that controls the cell cycle by regulating the cyclin-dependent kinases [7]. At the same time, *p21* is also one of the most studied downstream target genes of p53. Two highly conserved p53 responsive elements (p53REs) in the *p21* promoter region can be recognized and bound by activated p53 to activate *p21* gene expression [8]. We previously showed that *p21* expression is negatively regulated by the INO80 chromatin remodeling complex through binding to the p53REs in the *p21* promoter region [9]. In more detail, INO80 protein (a catalytic subunit of the INO80 complex) and YY1 (Yin Yang 1) (a core subunit of the INO80 complex) co-occupy with p53 at the p53RE sites of the *p21* promoter region in a p53-mediated mechanism. As a DNA-binding protein, YY1 contains both transcriptional activation and repression domains, thus showing a bidirectional function in gene transcription regulation [10,11]. Therefore, YY1 is widely involved in the transcriptional regulation of many intracellular genes. In cells, about 10% of all human genes contain YY1 binding motifs in their promoter regions [12]. Interestingly, the YY1 binding sequence (ACAT) appears in the center of p53RE sites of the *p21* promoter region [13]. Knockdown YY1 with siRNA results in p53 accumulation, and conversely, over-expression of YY1 promotes p53 degradation, suggesting that YY1 is a negative regulator of p53 [14].

BCCIP, a protein that is characterized based on its interaction with BRCA2 and CDKN1A (*p21*), not only directly binds to *p21* [15,16] but also connects with YY1. There are two different transcripts encoding BCCIPα (322 amino acids) and BCCIPβ (314 amino acids) in human cells. Both isoforms are composed of N-terminus acidic domain (NAD), internal conserved domain (ICD), and C-terminus variable domain (CVD) [15,16]. Interestingly, the NAD and ICD domain sequences in BCCIPα and BCCIPβ are identical [16]. Thus, the functional similarities between two isoforms can be surmised. Recent research data demonstrates that BCCIP maintains YY1 protein stability by directly binding to it in HCT116 cells. Co-transfection/coimmunoprecipitation (CoIP) experiments have confirmed that YY1/146-270 amino acids are the binding region for BCCIP, and at the same time, the BCCIP/ICD domain plays a key role in regulating YY1 stability through the ubiquitin-proteasome-mediated degradation pathway [17]. Based on a chromatin immunoprecipitation (ChIP)-Seq database search from the University of California Santa Cruz (UCSC) Genome Browser (http://genome.ucsc.edu), the enrichment of YY1 at the BCCIP transcriptional start site (TSS) proximal region in several cancer cells including A549 lung cancer cells, HepG2 human hepatocellular carcinoma cells, and HCT116 human colon cancer cells was found, suggesting the function of YY1 in regulating BCCIP gene transcription. This view is also confirmed by later experiments showing that YY1 and INO80 together transcriptionally regulate BCCIP in HCT16 cells [18]. However, transcriptional regulation of YY1 on BCCIP is in turn modulated by BCCIP itself in a YY1-dependent fashion [17]. On the other hand, stably expressing shBCCIP abrogates the transactivation activity of p53 and results in low expression of *p21*, suggesting the involvement of BCCIP in *p21* transcription [19,20]. Although YY1 and BCCIP are involved in the transcriptional regulation of p53, how the YY1/BCCIP complex coordinately regulates p53 and its target gene *p21* in cells is unclear. Therefore, in an effort to address this issue, using a series of biochemical and biological experiment approaches, we present evidence of the cross-talk between YY1, BCCIP, and p53. In addition, we investigated how YY1 and BCCIP coordinate with each other to regulate p53 and its target gene *p21*.

## 2. Results

### 2.1. Stabilization of BCCIP on p53 Was Regulated by YY1 in HCT116 (p53+/+) Cells

**Figure 1:**
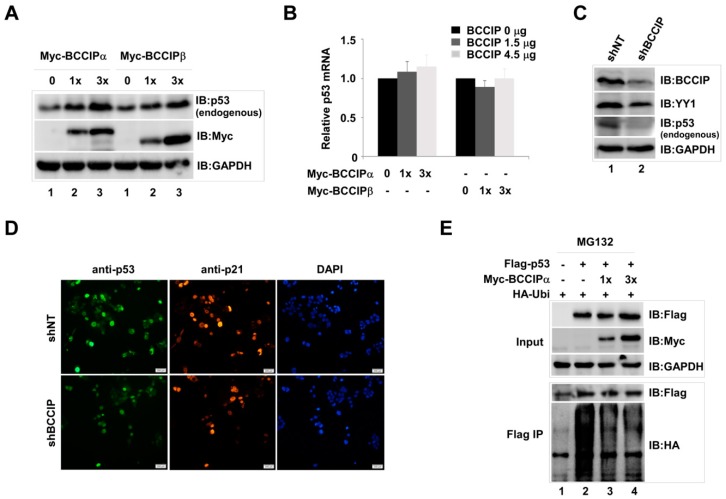
BCCIP stabilized p53 in HCT116 (p53+/+) cells. (**A**,**B**) Increase in endogenous p53 protein by transfection of BCCIP plasmids. Cells were transfected with 1.5 and 4.5 μg Myc tagged BCCIPα or BCCIPβ plasmids. Endogenous p53 protein in whole cell lysate and p53 mRNA levels were measured 72 h later with western blot methods (**A**, GAPDH is an internal control) and RT-qPCR (**B**, relative mRNA levels were normalized by GAPDH). (**C**,**D**) Decrease of endogenous p53 proteins in shBCCIP-treated cells. BCCIP shRNA (2 μg) and an shNT control was transfected into HCT116 cells. Indicated proteins were detected 72 h after transfection by western blot and cell staining. DAPI staining shows total nuclei. Scale bar indicates 200 µm. (**E**) Stabilization of exogenous p53 by transfection of BCCIPα plasmids. Cells were co-transfected with Flag-p53 (2.8 μg), Myc-BCCIPα (1.2 and 3.6 μg), and HA-ubiquitin (2 μg) in the presence of MG132 (10 μM). Exogenous p53 proteins were measured 48 h after transfection by western blot using anti-Flag antibody. The degradation of Flag-p53 was measured with anti-HA antibody.

In our preliminary experiments, we noticed that p53 protein expression levels were regulated by BCCIP. Thus, in order to confirm this phenomenon, we first examined the endogenous p53 protein level in Myc-BCCIP-expressing cells. The result, as shown in Figure 1A, endogenous p53 protein was dose-dependently increased in Myc-BCCIP-expressed cells at 72 h after transfection without affecting p53 mRNA levels (Figure 1B). To further explore the role of BCCIP in stabilizing p53, endogenous p53 protein was examined in pLVX-shNT (non-targeting shRNA) and pLVX-shBCCIP transfected cells. As expected, the endogenous p53 protein level in shBCCIP-expressing cells clearly decreased compared to the shNT group (Figure 1C, lane 2), suggesting the role of BCCIP in stabilizing endogenous p53 protein. Similarly, decreased endogenous p53 and its downstream target gene *p21* proteins were observed by fluorescent staining of shBCCIP-expressing HCT116 cells (Figure 1D). To further confirm the function of BCCIP in stabilizing p53, the effect of transient transfection of BCCIP on the level of exogenous p53 protein was detected in the presence of MG132 and HA-ubiquitin. Similar results were then observed in cells transfected with Flag-p53. Compared to the pcDNA3.1 vector (empty vector) group (Figure 1E, lane 1), higher p53 protein levels were observed at 48 h in the Myc-BCCIPα-transfected cells. Importantly, a dose-dependent decrease of ubiquitination was exhibited in BCCIP-transfected groups after Flag immunoprecipitation (IP), suggesting that BCCIP may regulate p53 stability through the ubiquitin-proteasome-mediated degradation pathway.

**Figure 2:**
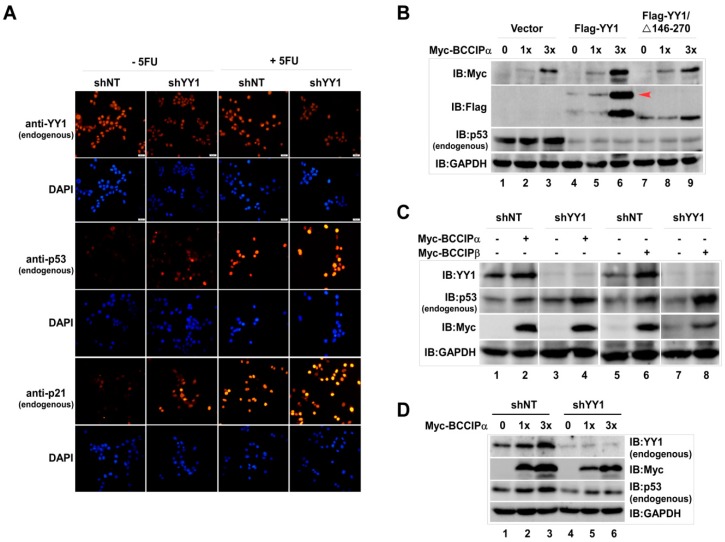
Stabilization of p53 by BCCIP was impacted by YY1 in HCT116 (p53+/+) cells. (**A**) Negative regulation of p53 on YY1. Cells were transiently transfected with shYY1 (12 μg/10 cm culture plate) and a shNT control for 72 h. Then, endogenous YY1, p53, and p21 proteins were immune-stained by indicated antibodies. Scale bar indicates 200 µm. (**B**) Impact of Flag-YY1 on p53-stabilization by BCCIP. Cells were co-transfected with Myc-BCCIPα (1.5 and 4.5 μg) and Flag-YY1 (2 μg) or deletion mutant Flag-YY1/∆146-270 (0.4 μg) for 48 h. Indicated proteins were analyzed by western blot with specific antibodies. (**C**) Impact of shYY1 on p53 stabilization by BCCIP. The experiments were designed as shown. Whole cell lysate was prepared and endogenous p53 protein levels were detected 72 h after shYY1 transfection using anti-p53 antibody. (**D**) Endogenous p53 protein levels in stably expressing shYY1 cells. Endogenous p53 protein was measured at 72 h after transfection with BCCIPα in stably expressing shYY1 cells.

On the other hand, according to our previous reports, BCCIP can also stabilize intracellular YY1 protein [17]. So, to know whether the stabilization effect of BCCIP on p53 is impacted by YY1, the effect of YY1 on p53 was first examined. In order to better visualize endogenous p53 by cell staining, fluorescent staining experiments were performed after treating cells with 5-Fluorouracil (5FU). Compared with the shNT group, it was clear that YY1 knockdown up-regulated p53 and its target gene *p21* expression, and this effect was more pronounced in 5FU-treated cells (Figure 2A). In contrast, compared to the empty vector group, endogenous p53 protein levels in BCCIP transfected cells were inhibited by transfecting Flag-tagged full-length YY1 and deletion mutant YY1/146–270 amino acids (YY1/∆146–270), which were the binding regions for BCCIP (Figure 2B, lanes 4–9/IB:p53). After confirming the negative regulation of YY1 on p53, an experiment with a design shown in Figure 2C was carried out. Consistent with previous results, endogenous p53 protein was increased by overexpressing BCCIP (lanes 2 and 6), and this increased level was further raised by transient knockdown of YY1 (lanes 4 and 8). However, in the stably expressing shYY1 cells, the dose-dependent increase of endogenous p53 by BCCIP was significantly suppressed (Figure 2D, lanes 5–6 in comparison with lanes 2–3), indicating the complexity of YY1/BCCIP in regulating endogenous p53 protein levels.

### 2.2. Cross-Interaction between BCCIP, YY1, and p53 Was Verified in HCT116 (p53+/+) Cells

**Figure 3:**
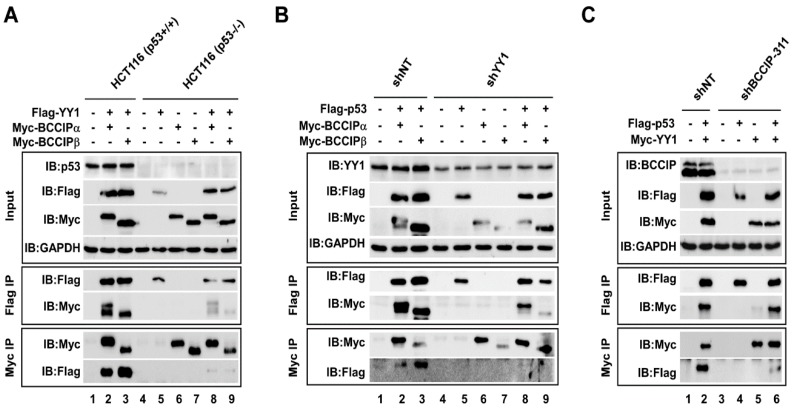
Cross-interaction between BCCIP, YY1, and p53 was verified in HCT116 (p53+/+) cells. (**A**) Interaction between BCCIP and YY1. In vitro co-transfection/coimmunoprecipitation (CoIP) experiments with anti-Flag M2 and anti-c-Myc affinity agarose were carried out in HCT116 (p53+/+) and HCT116 (p53−/−) whole-cell lysates. Bound proteins were visualized by western blot. (**B**) Interaction between p53 and BCCIP in shNT- and shYY1-treated HCT116 cells. CoIP experiments with anti-Flag M2 and anti-Myc antibodies were performed in HCT116 cells, and bound proteins were detected by western blot. (**C**) Interaction between p53 and YY1 in shNT- and shBCCIP-treated HCT116 cells. CoIP experiments with anti-Flag M2 and anti-Myc antibodies were done as designed.

The stabilizing effect of BCCIP on p53 combined with previous reports suggested that a cross-interaction between BCCIP, p53, and YY1 may exist. To address this issue, co-transfection/Co-IP experiments were performed. At first, the interaction between YY1 and BCCIP was tested in p53 wild type (p53+/+) and p53-null (p53−/−) HCT116 cells. As shown in Figure 3A, endogenous p53 could only be detected in HCT116 (p53+/+) cells (Input, IB:p53). After confirming the expression levels of Flag-YY1 and Myc-BCCIP (Input), Co-IP experiments were done using anti-Flag M2 and anti-c-Myc affinity agarose. It was obvious that BCCIP (both BCCIPα and BCCIPβ) and YY1 were bound each other (lanes 2–3, Flag IP and Myc IP), but in the case where p53 was absent, the binding ability of the two proteins was significantly reduced (lanes 8–9, Flag IP/IB:Myc and Myc IP/IB:Flag). Next, the interaction between p53 and BCCIP was determined in stably expressing pLVX-shNT and pLVX-shYY1 cells (Figure 3B). The YY1 knockdown efficiency is shown in the input panel (IB:YY1). Similarly, in cells lacking YY1, the binding ability of p53 and BCCIP was much weaker than in cells with normal expression levels of YY1 (lanes 8–9, Flag IP/IB:Myc and Myc IP/IB:Flag). It is worth noting that the binding activity between p53 and BCCIPβ (lane 9) was extremely low compared to that between p53 and BCCIPα (lane 8). The same co-transfection/Co-IP experiments were carried out in cells stably expressing pLVX-shNT and pLVX-shBCCIP (Figure 3C). The BCCIP knockdown efficiency is shown in the input panel (IB:BCCIP). Compared to the shNT group, the interaction between p53 and YY1 was decreased in cells lacking BCCIP (lane 6 compared to lane 2). Taken together, the protein–protein interaction between YY1, BCCIP, and p53 suggested the existence of an intrinsic link in biological function between three proteins.

### 2.3. BCCIP and YY1 Coordinate the p53 Responsive Element (RE) Mediated Luciferase Activity in HCT116 (p53+/+) Cells

**Figure 4:**
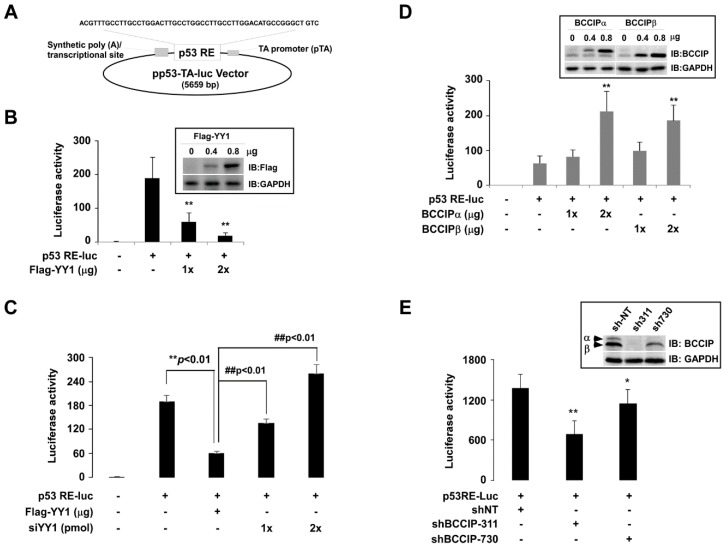
P53RE-mediated luciferase activity was regulated by YY1 and BCCIP in HCT116 (p53+/+) cells. (**A**) Schematic diagram of p53RE-Luc plasmid. Multiple response elements of p53 were inserted into a pp53-TA-Luc vector. (**B**,**C**) Effects of YY1 on p53RE-Luc luciferase activity. P53RE-Luc dual luciferase activity was measured after co-transfection with p53RE-Luc and Flag-YY1 or siYY1 (48 h) (*n* = 3). ** *p* < 0.01, *t*-test, comparison to p53RE-Luc-transfected group, while ## *p* < 0.01, *t*-test, comparison to Flag-YY1-transfected group. (**D**,**E**) Effects of BCCIPα/β on p53RE-Luc luciferase activity. P53RE-Luc dual luciferase activity was measured after co-transfection of p53RE-Luc and BCCIPα/β or shBCCIP (48 h) (*n* = 3). ** *p* < 0.01, *t*-test, comparison to p53RE-Luc-transfected group. shBCCIP-311 and shBCCIP-730 target different sequences.

To further explore the coordinative roles of BCCIP and YY1 in the regulation of the p53RE-mediated transcription of downstream target genes, multiple p53 responsive elements containing pp53-TA-Luc plasmid (p53RE-Luc) (Figure 4A) were used as a model to reflect p53 transcriptional activity. P53RE-Luc activity was determined by dual luciferase assay. Then, the impact of YY1 on p53 transactivation was estimated by co-transfection of p53RE-Luc with Flag-YY1 plasmids or YY1-specific siRNA. In contrast to basal-level luciferase activity, co-transfection of p53RE-Luc and YY1 dose-dependently decreased p53RE luciferase activity (Figure 4B, columns 3–4), and this effect, in turn, was reversed by simultaneous transfection of siYY1 (Figure 4C, columns 4–5). Exogenous Flag-YY1 proteins are shown in the upper right corner of Figure 4B. Next, we measured the effect of BCCIP on p53RE luciferase activity. In contrast to the effect of YY1, co-transfection of p53RE-Luc and BCCIP (both BCCIPα and BCCIPβ) dose-dependently increased p53RE luciferase activity compared the basal level (Figure 4D). However, the basal level of p53RE luciferase activity was inhibited by transfecting pLVX-shBCCIP (Figure 4E). Exogenous BCCIP proteins and BCCIP knockdown efficiency are shown in the upper right corners of Figure 4D and Figure 4E, respectively.

**Figure 5:**
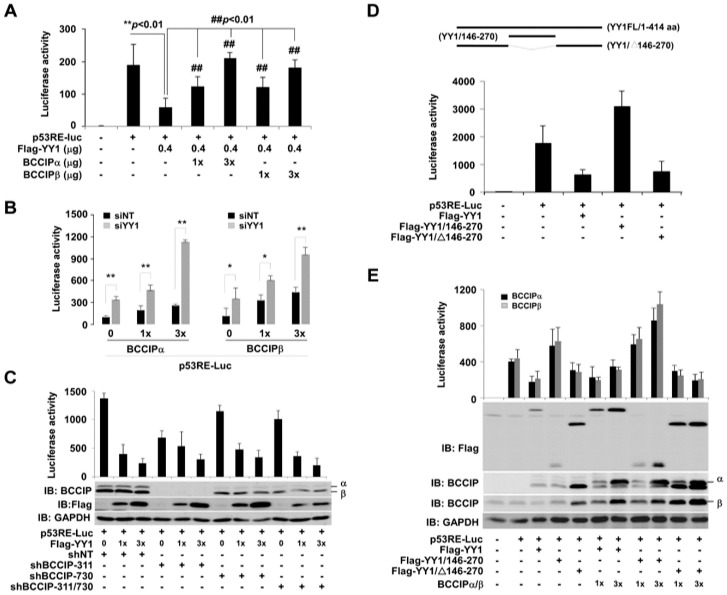
YY1 and BCCIP co-regulated the p53RE-mediated luciferase activity in HCT116 (p53+/+) cells. (**A**) Effects of BCCIP on p53RE-Luc activity in the Flag-YY1 transfected cells. Experiments were performed with the indicated design. ** *p*< 0.01, *t*-test, comparison to empty vector-transfected group, while ## *p* < 0.01, *t*-test, comparison to Flag-YY1-transfected group. (**B**) Effects of BCCIP on p53RE-Luc activity in the YY1 knockdown cells. Experiments were performed with the indicated design. ** *p* < 0.01, *t*-test, comparison to empty vector-transfected group. (**C**) Effect of YY1 on p53RE-Luc activity in the BCCIP knockdown cell. Experiments were performed with the indicated design. (**D**) Impact of deletion mutants of YY1 on p53RE-Luc activity. The upper panel shows a schematic diagram of deletion mutants of YY1. p53RE-Luc luciferase activities were measured after co-transfection of p53RE-Luc and deletion mutants of YY1 (48 h) (*n* = 3). ** *p* < 0.01, *t*-test, comparison to p53RE-Luc-transfected group. (**E**) Coordinative roles of BCCIP and YY1 on p53RE-Luc activity. Experiments were performed with the indicated design.

BCCIP and YY1 coregulation of p53RE luciferase activity was determined by co-transfection of p53RE-Luc with BCCIP and YY1. As shown in Figure 5A, decreased p53RE luciferase activity by YY1 was dose-dependently enhanced by transfecting both BCCIPα and BCCIPβ (^##^ *p* < 0.01, columns 4–7 in comparison with column 3). In contrast, the increase in p53RE luciferase activity due to exogenous BCCIPα and BCCIPβ was superimposed by knocking down YY1 (Figure 5B). Next, p53RE luciferase activity was measured by co-transfection of Flag-YY1 (0.3 and 0.9 µg) in the stably expressing pLVX-shBCCIP cells. shBCCIP-311 and shBCCIP-730 represent two different targeting sequences for BCCIP. The results were shown in Figure 5C. No matter which sequence region was targeted, compared to shNT groups, knocking down BCCIP appeared to result in no big changes in p53RE luciferase activity reduction due to YY1. Based on our previous report [17], the 146–270 amino acids of YY1 are the binding region for BCCIP. We therefore designed experiments as shown in Figure 5D. The upper panel presents the deletion mutants of YY1. As a result, the truncated YY1 (YY1/146–270 amino acids) showed an activity opposite to that of the full-length YY1 (column 4 compared to column 3). Interestingly, the p53RE luciferase activity of YY1-lacking residues 146–270 (YY1/Δ146–270) remained the same as that of the full-length YY1 (columns 3 and 5), indicating that the region 146–270 amino acids of YY1 may be related to the transcriptional activation of YY1. Similar experiments were then performed with or without BCCIP transfection (Figure 5E). In the absence of BCCIP, the impact of both BCCIPα and BCCIPβ on p53RE luciferase activity (columns 2–5) was similar to Figure 5D. There was a slight increase for the BCCIP-transfected group (column 7) compared to the full-length YY1-transfection group. In addition, an additive effect between BCCIP and the YY1/146–270 region on p53RE luciferase activity was observed (column 9 in comparison with column 4). However, BCCIP did not change the effect of deletion mutant YY1 (YY1/∆146–270) on p53RE luciferase activity (columns 10 and 11 in comparison with column 5).

### 2.4. P53 Downstream Target Gene p21 Was Coordinately Regulated by the YY1/BCCIP Complex in HCT116 (p53+/+) Cells

**Figure 6:**
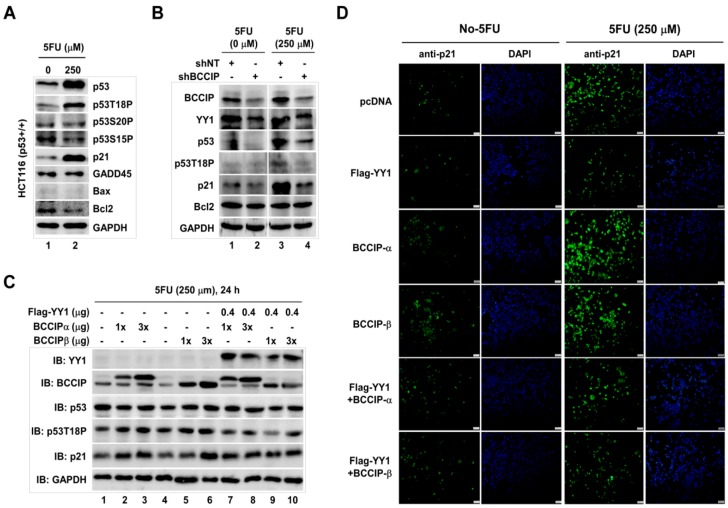
P53 downstream target gene *p21* was coordinately regulated by YY1 and BCCIP in HCT116 (p53+/+) cells. (**A**) Induction of p53 by 250 µM 5FU. Indicated proteins were analyzed by western blot with specific antibodies. (**B**) Impact of shBCCIP on 5FU-induced p53. shNT- and shBCCIP-transfected cells were treated with 0 and 250 µM 5FU, and indicated proteins were measured by western blot with antibodies. (**C**) Coordinative effects of YY1 and BCCIP on p53. The experiment was designed as shown. Proteins in prepared lysate were analyzed by western blot. (**D**) Immune staining. Cells were transfected with indicated plasmids in the presence or absence of 5FU. Then, cells were stained with anti-p21 antibody. DAPI staining shows total nuclei. Scale bar indicates 200 µm.

A classic chemotherapeutic drug, 5-Fluorouracil (5FU), has been shown to induce p53 [21]. In our experiment conditions, p53/p53T18P (threonine 18 site phosphorylated p53) and its target gene *p21*, but not GADD45/Bax/Bcl2, were significantly induced by 250 µM 5FU (Figure 6A). However, this induction was blocked in the pLVX-shBCCIP-transfected cells (Figure 6B, lane 4). In addition, consistent with previous reports [17], YY1 protein levels significantly declined in BCCIP knockdown cells with or without 5FU treatment (Figure 6B, lane 2/IB:YY1). Next, the coordinative effects of BCCIP/YY1 on p53 and p21 protein levels were examined by western blot in 250 µM 5FU-exposed HCT116 cells. The results, as shown in Figure 6C, indicate a dose-dependent increase of p53T18P and p21 in BCCIP-transfected cells in the presence of 5FU (lanes 2–3 and lanes 5–6 compared to lane 1 and 4, respectively). However, this phenomenon disappeared by co-transfecting BCCIP with YY1 (lanes 7–10). Protein levels were also visualized by cell staining after co-transfection of BCCIP and YY1 in the presence or absence of 5FU cells (Figure 6D). Similar to western blot results, induction of p21 was observed in 5FU-exposed cells. This induction was enhanced by BCCIP transfection. However, it was suppressed by co-transfecting YY1, suggesting the collaborative work of BCCIP/YY1 on p53 and its target gene *p21*.

### 2.5. Coregulation of the YY1/BCCIP Complex on p53 Target Gene p21 Was Clarified in HCT116 (p53+/+) Cells

**Figure 7:**
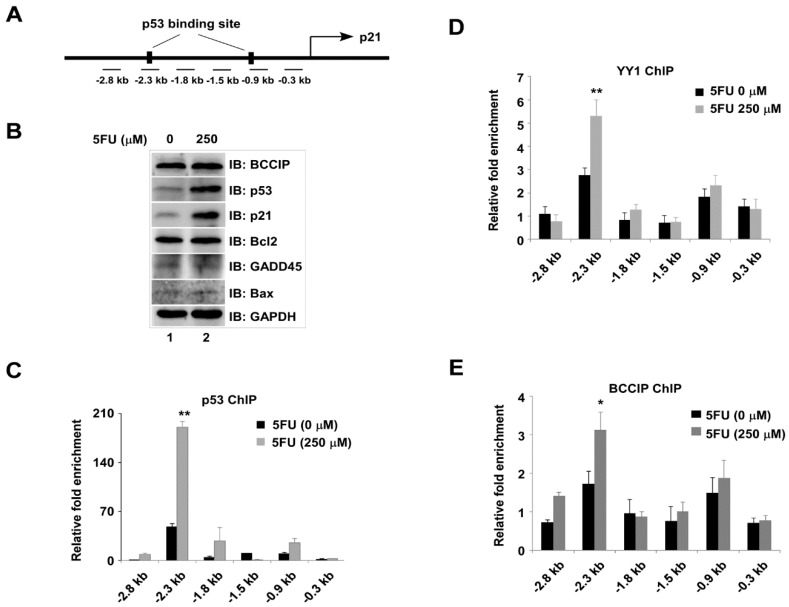
Co-occupancy of YY1, BCCIP, and p53 at p53RE sites in *p21* gene in HCT116 cells. (**A**) Six primer sets at the *p21* locus designed for amplifying chromatin immunoprecipitation (ChIP) DNA. Chromatin lysates were prepared from cells with or without 5FU treatment. 5FU-induced proteins were analyzed by western blot (**B**). ChIP experiments were performed using anti-p53 (**C**), anti-YY1 (**D**), and anti-BCCIP (**E**) antibodies. ChIP DNA amplified with qPCR. The y-axis shows the ratio of ChIP DNA signals to IgG, and all signals were normalized to input (*n* = 3). * *p* < 0.05, ** *p* < 0.01, *t*-test, comparison to 5FU untreated group.

**Figure 8:**
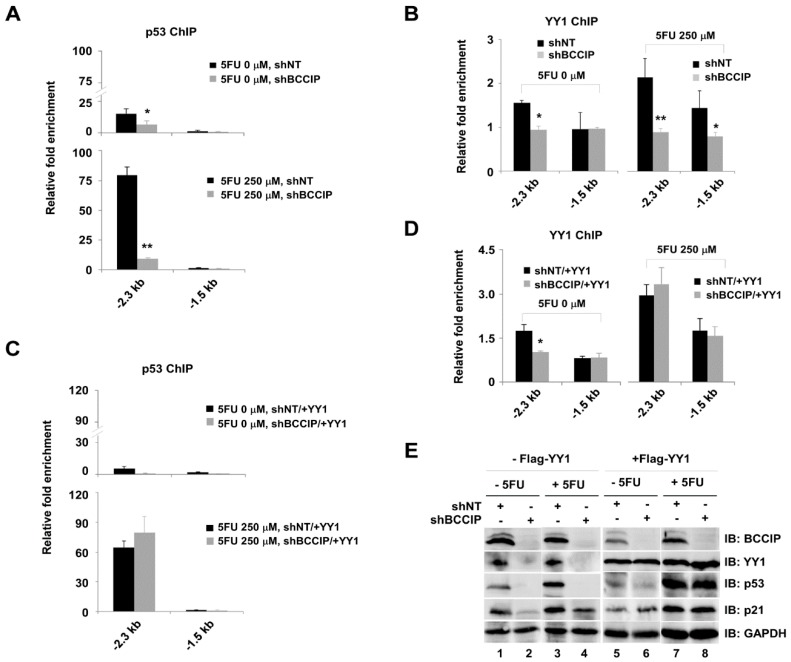
YY1 and BCCIP coregulation of p53 target gene *p21* in shBCCIP-treated HCT116 cells. BCCIP knockdown cells with or without 5FU exposure were used in the following ChIP experiments. (**A**,**B**) p53 and YY1 ChIP experiments were carried out using chromatin lysate in the presence of shNT or shBCCIP expressing cells. Chip DNA was analyzed with qPCR (*n* = 3). (**C**,**D**) Rescue experiments. Stably expressing shNT or shBCCIP cells were transiently transfected with Flag-YY1 for 48 h. Then, prepared chromatin lysate was applied to ChIP assays. The y-axis shows the ratio of ChIP DNA signals to IgG, and all signals were normalized to input (*n* = 3). * *p* < 0.05, ** *p* < 0.01, *t*-test, comparison to shNT group. (**E**) Indicated protein levels in A, B, C and D.

In order to further confirm the coordinative effect of BCCIP/YY1 on p53 and its target gene *p21*, six primer sets including p53 binding sites in the *p21* locus were used to amplify ChIP DNA (Figure 7A). Prepared chromatin lysate from 0 or 250 µM 5FU-treated cells were applied for p53, YY1, and BCCIP ChIP assays. P53 and p21 proteins induced by 5FU were analyzed by western blot with specific antibodies (Figure 7B). It was clear that p53 (Figure 7C), YY1 (Figure 7D), and BCCIP (Figure 7E) were co-occupied at −2.3 kb upstream of the *p21* transcriptional start site (TSS). The enrichment of three proteins at −2.3 kb upstream of the *p21* locus in 5FU-treated cells was more obvious. Although not very strong, we can see the recruitment of p53 at −1.8 kb and p53/YY1/BCCIP at −0.9 kb upstream of the *p21* TSS. From the above experimental results, the existence of cross-talk between p53, YY1, and BCCIP in the transcriptional regulation of *p21* can be speculated. To address this speculation, ChIP assays were further carried out in stably expressing shBCCIP cells with or without 5FU treatment. Select primer sets at the *p21* locus including -2.3 kb and -1.5 kb were used to amplify ChIP DNA. As expected, silencing BCCIP prevented the recruitment of both p53 and YY1 at −2.3 kb upstream of the *p21* TSS compared to the shNT group (Figure 8A,B). Interestingly, transient transfection of YY1 restored the recruitment of p53 and YY1 at −2.3 kb upstream of the *p21* TSS (Figure 8C,D). However, no binding peaks of p53 and YY1 were seen at the non-p53 binding site (−1.5 kb). Protein levels, including all of the experimental conditions in Figure 8, are shown in E.

## 3. Discussion

In general, tumor suppressor gene *p53* appears inactivated in many cancers. However, *p53* can respond to various stimuli as a cellular stress sensor that triggers cell cycle arrest, apoptosis, DNA damage caused by various chemotherapeutic drugs, hypoxia, oxidative stress, and nutrient starvation periods [5,22]. Based on specific cellular responses, p53 transactivates and induces particular p53-mediated target genes such as *p21* [23,24]. Like other transcription factors, p53 contains specific domains that are responsible for sequence-specific DNA binding and transcriptional activation [25]. It has been reported that activated p53 can recognize and bind directly to two highly conserved p53REs in the *p21* promoter and induce transcriptional activation of the *p21* gene [8]. Using Re-ChIP experiments, we previously provided evidence that the INO80 chromatin remodeling enzyme in conjunction with p53 co-occupies at both –2.2kb and –1.0kb upstream of the *p21* TSS and negatively regulates *p21* expression in a p53-mediated mechanism [9]. It is worth noting that as an essential co-activator and a subunit of the INO80 complex, YY1 can recruit the INO80 complex to some target genes such as CDC6 and GRP78 [26]. Our data, combined with previously reported data, have confirmed that YY1 can be recruited at the p53 binding site in the *p21* promoter and be involved in the regulation of *p21* transcription [9,13], suggesting that the recruitment of INO80 complex to the *p53* binding site of the *p21* TSS may be achieved by YY1. Based on the most recent report, the recruitment of INO80 complex to the *p53* binding site in the *p21* promoter may function in two different ways according to the intracellular condition: i) the INO80 complex represses *p21* expression by regulating the promoter proximal nucleosome arrangement under normal cellular conditions; ii) in Doxo-treated cells, the INO80 complex rapidly removes accumulated H2A.Z and relieves its inhibition of *p21*, thereby inducing the transcriptional expression of *p21* [27].

On the other hand, we recently found that BCCIP not only stabilizes YY1 protein, but also directly binds to YY1 in HCT116 cells. The 146–270 amino acid of YY1 seemed to be the key region for maintaining the interaction between YY1 and BCCIP [17]. However, the interaction between the two proteins was extremely weakened in p53-null HCT116 cells (Figure 3A), demonstrating that the intracellular function of the YY1/BCCIP complex required the presence of p53 in cells. What is more, in our experimental conditions, p53 can bind to YY1 and BCCIP, respectively, but once YY1 or BCCIP was knocked down, the binding activity of p53 with the other protein was decreased, indicating the cross-interaction between p53, YY1, and BCCIP in cells. It is well known that the stability of the p53 protein directly affects the transcriptional activation of downstream target genes [28]. Given that the stabilization of p53 was regulated by both BCCIP and YY1, the coordinative function between p53, BCCIP, and YY1 on p53RE-mediated downstream target gene transcription, including *p21,* was surmised. Consistent with this, the luciferase activity of p53RE-Luc, which contains multiple p53REs, was regulated by both YY1 and BCCIP. As previously reported [14,19], YY1 inhibited p53RE-mediated transcriptional activation, whereas BCCIP revealed the opposite effect. Therefore, we can imagine that under normal circumstances, p53/YY1/BCCIP may maintain a dynamic balance by restricting each other. Any increase or decrease in either side can break this balance and affect the transcriptional activity of *p53*.

**Figure 9:**
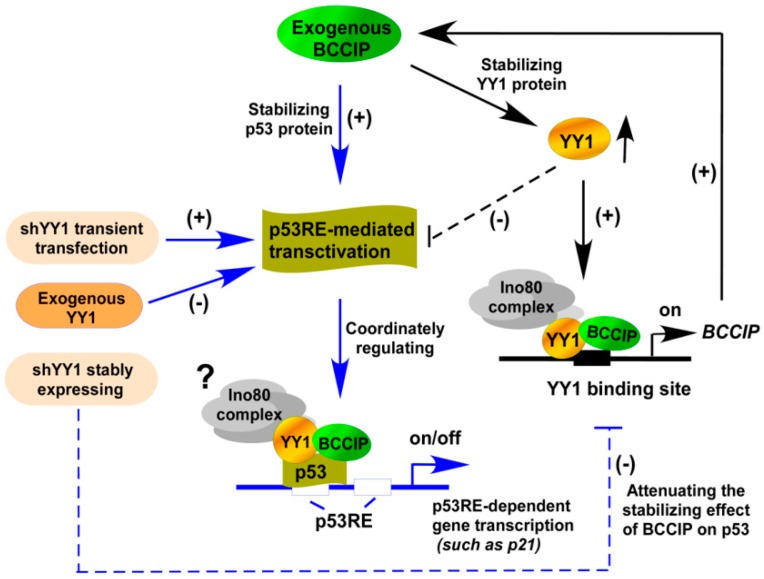
Coordinated regulation of p53RE-dependent gene transcription by p53/BCCIP/YY1. BCCIP promotes p53RE-mediated transactivation by binding to and stabilizing p53 and further activates *p21* transcription. This pathway is regulated by intracellular YY1 protein levels. Increased YY1 inhibits p53RE-mediated transactivation, and the opposite effect is seen in transient knockdown of YY1. However, stable knockdown of YY1 attenuates BCCIP transcription, further affecting p53 stability, which suggests that there may be feedback inhibition. Black solid arrows, published data [17]; black T dotted arrow, indirect inhibition; blue solid arrows, possible pathway of p53RE-dependent gene transcription; blue T dotted arrow, stably expressing shYY1 affect the INO80/YY1-BCCIP pathway.

Given that YY1 contains both transcriptional activation and repression domains, it can activate or repress gene transcription by recruiting different transcriptional cofactors to its activation or repression domain [10,29]. Our data supports this view. Contrary to the effect of full-length YY1, 146–270 amino acids of YY1 increased p53RE-mediated transcriptional activation. However, the deletion mutants YY1/∆146-270 maintained the same p53RE luciferase activities as the full-length YY1, suggesting that this region of YY1 may be involved in p53RE-mediated transcriptional activation. Once this region is blocked by interacting proteins, such as BCCIP, YY1 functions as a transcription inhibitor. We mentioned earlier that the binding ability of YY1 and BCCIP was significantly attenuated in p53-null HCT116 cells. In contrast, YY1/BCCIP complex affected p53 stability in HCT116 (p53+/+) cells. Interestingly, the effect of YY1/BCCIP complex on p53 was strongly related to the knockdown level of YY1. In the case of transiently knocking down YY1 and simultaneously transfecting BCCIP, the p53 protein level was increased (Figure 2C). However, when stably knocking down YY1 and then transfecting BCCIP, the p53 protein level in cells declined (Figure 2D). In another case, exogenous BCCIP dose-dependently increased p53RE-Luc-mediated *p21* and p53T18P expression levels, suggesting that p53 phosphorylation increases the transcriptional activity of p53 by enhancing p53 stability [28]. More in-depth research confirmed that p53 co-localized with YY1 and BCCIP at p53RE sites in the *p21* promoter, suggesting the coordinative roles of YY1/BCCIP/p53 in regulating p53RE-mediated *p21* transcription (Figure 9). For example, the binding of p53 and YY1 to the p53RE sites in *p21* was reduced by knocking down BCCIP, but this phenomenon was rescued by over-expressing YY1.

## 4. Materials and Methods

### 4.1. Antibodies

Anti-Flag (M2) and anti-c-Myc agarose, as well as anti-Flag M2 (F3165) mouse monoclonal antibody were purchased from Sigma (St. Louis, MO, USA). Anti-BCCIP (16043-1-AP) and anti-p21 (10355-1-AP) polyclonal antibodies were from Proteintech Group (Wuhan, China). Anti-YY1 (H414) (sc-1703 or sc-1703X) rabbit polyclonal antibody, anti-Myc (9E10, sc-40) mouse monoclonal antibody, rabbit total IgG (sc-2027), and mouse total IgG (sc-2025) were obtained from Santa Cruz Biotechnology (Dallas, TX, USA). Anti-p53 mouse monoclonal antibody was provided by Boster Group (BM0101, Wuhan, China). The ChIP grade anti-p53 mouse monoclonal antibody was from Abcam (ab1101, USA). Anti-p53S15P (RLP0205), anti-p53T18P (RLT0212), anti-p53S20P (RLT0206), anti-GADD45 (RLT1832), anti-Bax (RLT0456), and anti-Flag (RLG0004) rabbit polyclonal antibodies, Bcl2 (RLM3041) and anti-HA (RLM3003) mouse monoclonal antibodies were obtained from Ruiying (Suzhou, China). Anti-GAPDH (NM_002046, full length) polyclonal antibody and anti-BCCIP (NM_078468, residues 1-322) mouse polyclonal antibody were raised against bacterially expressed proteins (Jilin University, Changchun, China). 5-Fluorouracil (5FU) (F6627, Sigma) was dissolved in dimethyl sulfoxide (DMSO) and prepared at a 500 mM concentration for storage. The final concentration of 5FU in cell culture medium was 250 μM.

### 4.2. Cell Culture/Maintenance

The human HCT116 colon carcinoma cells including p53 wild type (p53+/+) or p53-null (p53−/−) and the human embryonic kidney (HEK) 293T cells were cultured in RPMI 1640 Medium (Gibco Life Technologies™, Gaithersburg, MD, USA.) and Dulbecco’s Modified Eagle’s Medium (Gibco Life Technologies™, Gaithersburg, MD, USA), respectively, containing 10% fetal bovine serum (KangYuan Biology, China) and a 1% penicillin–streptomycin mixture (Thermo Fisher Scientific, Waltham, MA, USA), at 37 °C in the presence of 5% CO_2_.

### 4.3. Co-Immunoprecipitation (Co-IP)

Whole-cell lysate was prepared 48 h after transient transfection plasmids. The method was described previously [17]. Bound proteins were eluted by 0.2 mg/mL peptides (Flag, Myc, or HA) or by SDS-PAGE loading buffer and detected with western blot analysis using specific antibodies.

### 4.4. Constructions of Plasmids and Transient Transfection

Full-length cDNAs encoding human BCCIP (NM_078468), p53 (NM_001276760), YY1 (NM_003403), and different truncations of YY1 including YY1 (146–270aa and YY1/Δ146–270aa) proteins were cloned with Flag and Myc tags into pcDNA3.1 (−). Tagged YY1, BCCIPα/β, and p53 plasmids (pcDNA3.1 as control) were transiently transfected to HCT116 cells using 4 µL PEI (23966, Polysciences, Beijing, China) according to the manufacturer’s recommendations. Cells were harvested and lysed 48 h after transfection. The proteins were analyzed by western blot with specific antibodies.

### 4.5. Luciferase Reporter Assay

Pp53-TA-Luc plasmid (D2223, Beyotime) including multiple p53 response elements (ACGTTTGCCTTGCCTGGACTTGCCTGGCCTTGCCTTGGACATGCCCGGGCTGTC) was obtained from Beyotime Biotechnology (Shanghai, China). HCT116 cells were co-transfected with Pp53-TA-Luc (0.4 μg), which encodes firefly luciferase, the control plasmid renilla luciferase vector (0.12 ng), which encodes renilla luciferase, and the plasmids expressing YY1 and BCCIP using PEI reagent (Polysciences, Beijing, China). Total effector plasmid (1.7 μg) in each transfection was adjusted with empty vectors. pp53-TA-Luc transactivity was estimated 48 h later by measuring firefly and renilla luciferase activities using the Dual-Luciferase reporter assay kit (Promega, Madison, WI, USA) and by normalizing firefly to Renilla luciferase [18].

### 4.6. Reverse Transcription PCR

Isolation of total RNA and reverse transcription (cDNA synthesis) were done using RNAiso Plus (D9109) (Takara, Tokyo, Japan) and PrimeScript 1st Strand cDNA Synthesis Kit (Takara) essentially as described [18]. P53 and GAPDH mRNA were measured by quantitative real-time PCR (qPCR) with an Eco™ Real-Time PCR System (Illumina, Gene Company Limited, Hongkong). The program of PCR reactions were finished as previously described [18]. The specific mRNA was measured by qPCR with the following RT-primer sets: p53, 5′-CAGCACATGACGGAGGTTGT-3’ (forward) and 5′-TCATCCAAATACTCCACACGC-3′ (reverse); GAPDH, 5′-ATCACTGCCACCCAGAAGAC-3′ (forward) and 5′-ATGAGGTCCACCACCCTGTT-3′ (reverse).

### 4.7. shRNA Knockdown

To knock down BCCIP or YY1 expression, the lentivirus system was used to express shRNA [19,21]. According to the previous report [19], the following shRNA sequences targeted at the shared region of BCCIP and YY1 were used: shBCCIP-311, 5′-GTGTGATTAAGCAAACGGATG-3′ and shBCCIP-730, 5′-GCTGCGTTAATGTTTGCAAAT-3′; shYY1-1, 5′-GGGAGCAGAAGCAGGTGCAGAT-3′; sh-YY1-3 5′-GATGCTGATGTTCAGTGTAATT-3′. All shRNA sequences were introduced into the pLVX vector. However, in siRNA interference experiments, 10 pmol siYY1 (sc-36863) (customized) and non-targeting siRNA (shNT, D-001206 as control) were used for knocking down YY1. Prepared whole-cell lysate was subjected to SDS-PAGE gel. The proteins were analyzed by western blot with specific antibodies.

### 4.8. Immunofluorescence Staining

About 30% cultured HCT116 cells in 24-well plates containing a cover slip (8D1007, Wuxi Nest Biotechnology Co., LTD., Jiangsu, China) on each well were treated with shBCCIP or shYY1 for 72 h. Cells were then washed, fixed with 4% paraformaldehyde for 15 min at room temperature, and permeabilized with 0.3% TritonX-100 in PBS buffer for 5 min. After incubating cells with p53 (1:50), p21 (1:100), and YY1 (1:100) primary antibodies at 37 °C for an hour, cells were stained with FITC- or TRITC-conjugated secondary antibodies (rabbit/green: 1:300, ZF-0311; rabbit/red: 1:300, ZF-0316; mouse/green: 1:300, ZF-0312; mouse/red: 1:300, ZF-0313). Cell nuclei were stained by DAPI containing Vectashield (Vector Laboratories, Inc. H-1200). Fluorescence images were observed with an Olympus BX40F microscope (Olympus Corporation, Tokyo, Japan).

### 4.9. Chromatin Immunoprecipitation (ChIP) Assay

ChIP assays were carried out essentially as described using p53, BCCIP, and YY1 antibodies [17]. Each ChIP used 2 × 10^7^ HCT116 cells. ChIP DNA was analyzed by quantitative real-time PCR (qPCR). Each experiment was performed 2–3 independent times. ChIP and no antibody signal were normalized to total input. Six primer sets for qPCR on the promoter region of *p21* were designed: *p21* −2.8kb (−2876bp~−2470bp), 5′-TGATGCTAGGAACATGAGCAA-3′ (forward) and 5′-CCCGAGTAGCTGGGATTACA-3′ (reverse); −2.3kb (−2292bp~−2111bp), 5′-CTGTGGCTCTGATTGGCTTT-3′ (forward) and 5′-CTCCTACCATCCCCTTCCTC-3′ (reverse); −1.8kb (−1876bp~−1788bp), 5′-ACATTCAAGTGCATGGTTGC-3′ (forward) and 5′-CTTCTAGCTCACCACCACCA-3′ (reverse); -1.5kb (−1511bp~−1326bp), 5′-GCTTAGAGTGGGGTCCTGAG-3′ (forward) and 5′-CCTCTAACGCAGCTGACCTC-3′ (reverse); -0.9kb (−982bp~−848bp); 5′-TTGTCATTTTGGAGCCACAG-3′ (forward) and 5′-GGGCTCAGAGAAGTCTGGTG-3′ (reverse); -0.3kb (−293bp~−193bp), 5′-GGGGCTCATTCTAACAGTGC-3′ (forward) and 5′-GACACATTTCCCCACGAAGT-3′ (reverse).

### 4.10. Statistical Analysis

Results are expressed as the means and standard deviation (SD). Comparisons between two groups were made with an unpaired Student’s *t*-test.

## 5. Conclusions

In summary, our data suggest that the YY1/BCCIP complex coordinatively regulates p53RE-mediated *p21* transactivation. Also, the cross-interaction between YY1, BCCIP, and p53 indicates the intrinsic link between three proteins in the regulation of *p21* transcription. These data provide new insights into the transcriptional regulation of *p21* by the YY1/BCCIP complex.

## Abbreviations

CDKN1A (p21)	Cyclin-dependent kinase inhibitor 1A
p53RE	p53 responsive elements
BCCIP	BRCA2 and CDKN1A-interacting protein
YY1	Yin Yang 1
5FU	5-Fluorouracil
TSS	transcriptional start site.
